# Supplementary material for: A sustained change in the supply of parental care causes adaptive evolution of offspring morphology
Source: Nat Commun. 2018 Sep 28;9:3987. doi: 10.1038/s41467-018-06513-6 (PMC6162320; doi:10.1038/s41467-018-06513-6)
Supplement: Supplementary file 1 — Supplementary Information [file 41467_2018_6513_MOESM1_ESM.pdf]

**Supplementary Information**

**A sustained change in the supply of parental care causes adaptive evolution of offspring morphology**

**Jarrett et al.**

**Supplementary Figures**

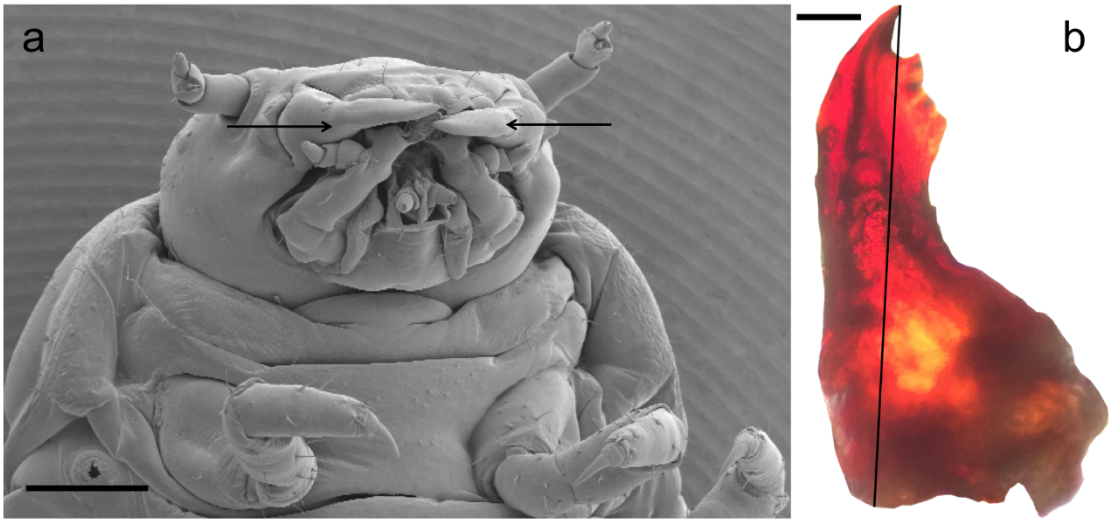


**Supplementary Figure 1**: **a** The head of a burying beetle larva. The two arrows show the two mandibles. The scale bar in the bottom left equals 500μm. Scanning electron microscope image courtesy of Claudia Grossman.

**b** A dissected and mounted mandible. The length measured is shown with the black line. The scale bar in the top left corner equals 90μm.

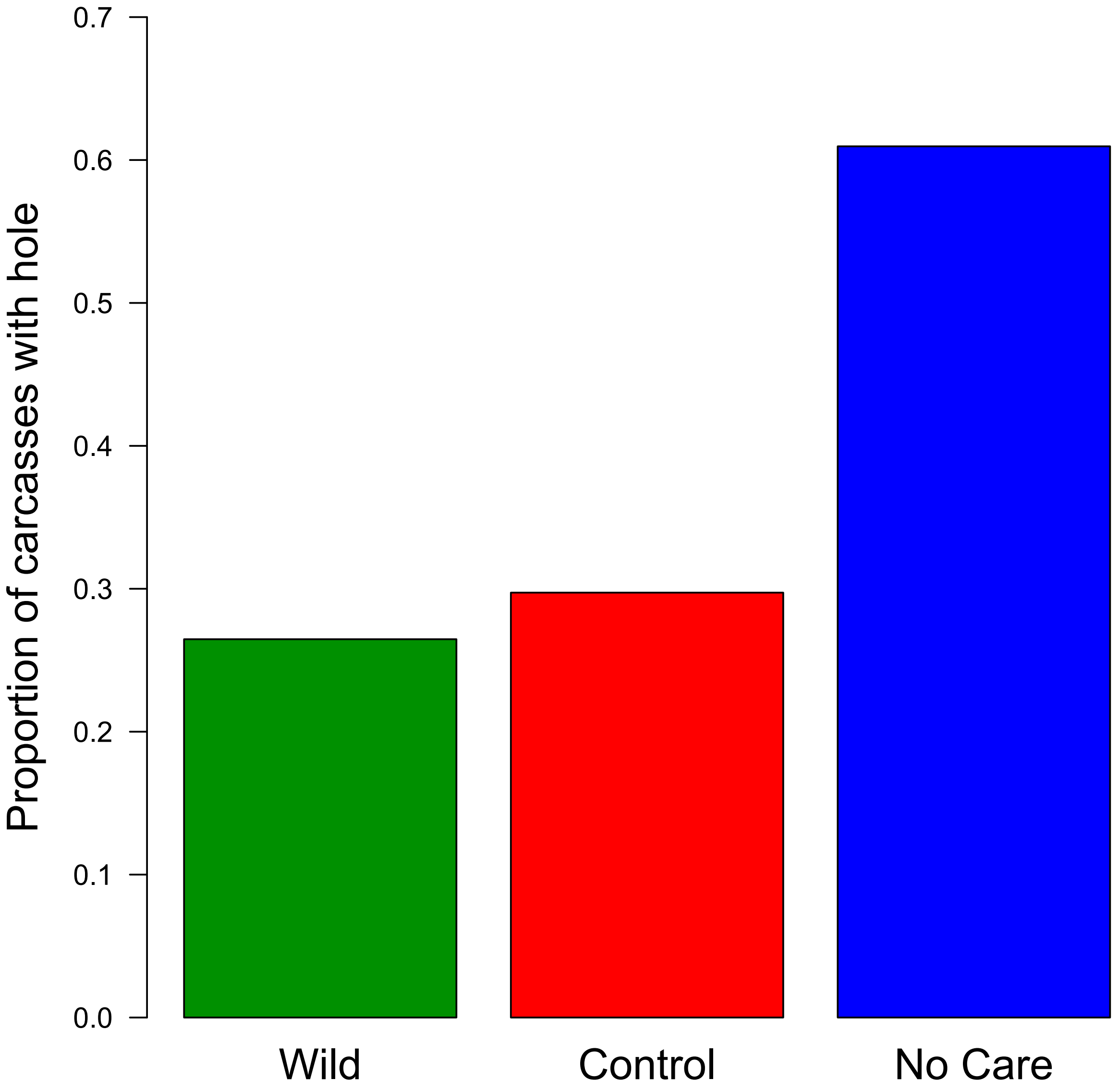


**Supplementary Figure 2:** The proportion of breeding pairs that made an incision in the carcass, ~53 hours after being paired (i.e prior to larval hatching), for beetles drawn from wild population, and the replicate experimental populations evolving in a Control environment or under a No Care environment. Note that the data for the wild-caught population are exactly the same as those depicted in Fig. 1. The proportion of carcasses where a hole was inserted prior to hatching are: Wild = 9/34; Control treatment = 44/148 ($1^{st}$ replicate population = 20/74, $2^{nd}$ replicate population = 24/74); and No Care = 89/146 ($1^{st}$ replicate population = 43/72, $2^{nd}$ replicate population = 46/74).

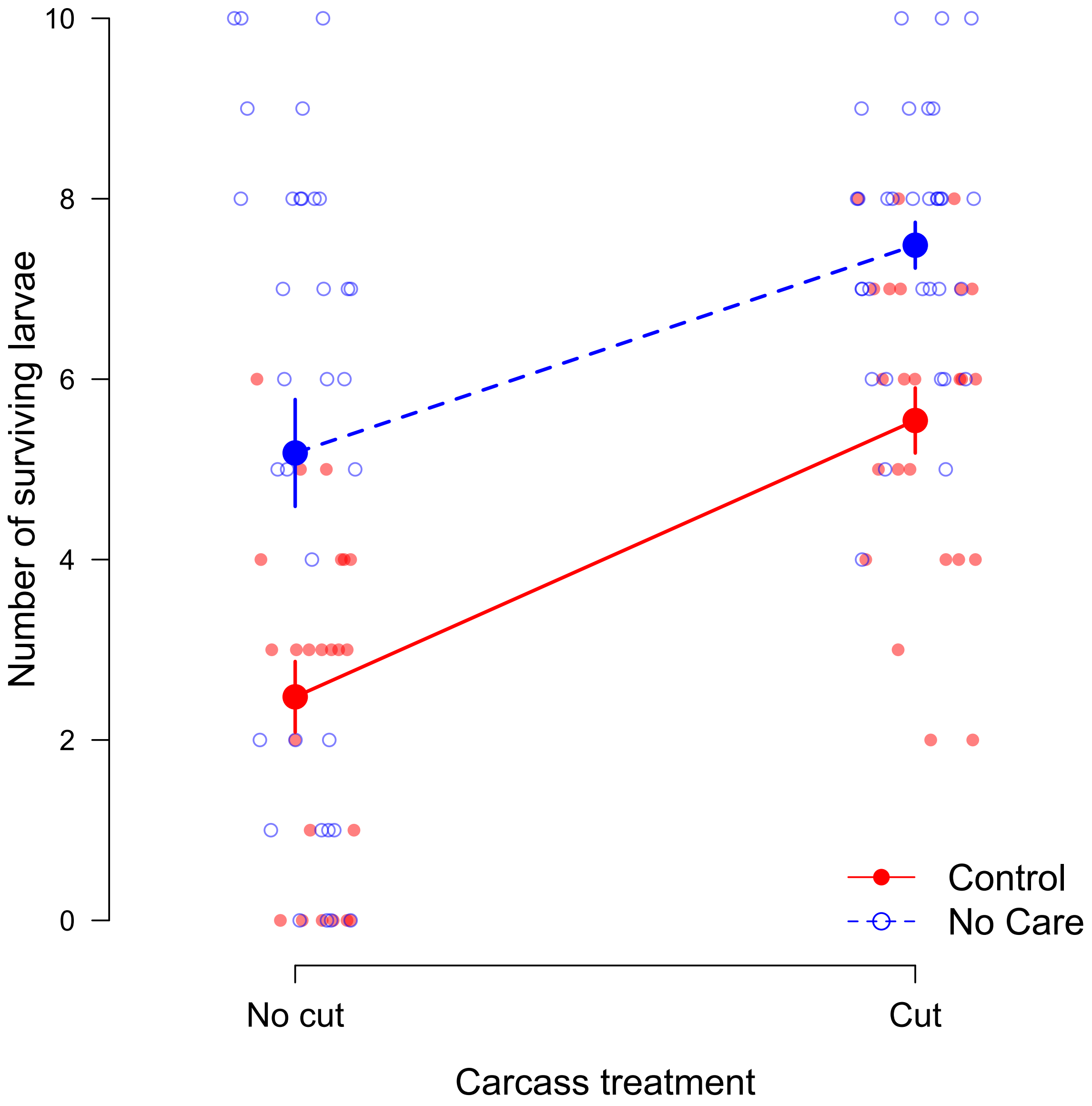


**Supplementary Figure 3:** The number of larvae surviving without post-hatching care, from initial experimental broods of 10 larvae, when an incision was experimentally added to a carcass prepared by stock beetles (Cut) and when it was not (Uncut). Data for larvae drawn from the Control replicate populations are shown in red, while data for larvae from the No Care replicate populations are shown in blue. Control Uncut: N = 23; Control Cut: N = 24; No Care Uncut: N = 33; No Care Cut: N = 33. Means and standard errors are shown, connected by reaction norms.

## Supplementary Tables

**Supplementary Table 1:** The allometric slopes (β) of the relationship between larval mass and the cube of mandible length of wild larvae in two social environments: Full Care and No Care. Numbers in brackets refer to lower and upper 95% confidence limits, respectively. The Combined row presents equivalent statistics when data from both populations are pooled. Both ordinary least squares (OLS) and major axis (MA) regression estimates are shown.

| Environment | OLS | MA |
|---|---|---|
| Full Care<br>(N = 54) | 0.126<br>(-0.032, 0.283) | 0.183<br>(-0.049, 0.435) |
| No Care<br>(N = 54) | -0.009<br>(-0.158, 0.141) | -0.012<br>(-0.234, 0.208) |
| Combined<br>(N = 108) | 0.088<br>(-0.022, 0.198) | 0.130<br>(-0.034, 0.301) |

**Supplementary Table 2:** The allometric slopes (β) of the relationship between larval mass and the cube of mandible length of larvae that have evolved under two different social environments: Full Care and No Care, combining data from the replicate populations (F1 and F2 combined for Full Care, N1 and N2 combined for No Care). Numbers in brackets refer to lower and upper 95% confidence limits, respectively. Both ordinary least squares (OLS) and major axis (MA) regression estimates are shown.

| Population | OLS | MA |
|---|---|---|
| Full Care<br>(F1 = 45, F2 = 37) | 0.382<br>(0.315, 0.448) | 0.414<br>(0.343, 0.488) |
| No Care<br>(N1 = 46, N2 = 39) | 0.266<br>(0.220, 0.312) | 0.278<br>(0.230, 0.326) |
